# Molecular Acrobats: How CHD Remodelers Shape the Genetic Playground to License Cell Identity

**DOI:** 10.1002/bies.70158

**Published:** 2026-07-13

**Authors:** İsa Özdemir, Sarah J. Hainer

**Affiliations:** ^1^ Department of Biological Sciences University of Pittsburgh Pittsburgh Pennsylvania USA; ^2^ UPMC Hillman Cancer Center University of Pittsburgh Pittsburgh Pennsylvania USA

## Abstract

In eukaryotes, DNA is not a naked repository of genetic information but is tightly wound around histone octamers to form nucleosomes. While this packaging solves the spatial problem of fitting 2 meters of DNA into a microscopic nucleus, it creates a massive accessibility problem for DNA‐templated events, including transcription, replication, and DNA repair. Here, we review how the chromodomain helicase DNA‐binding (CHD) family of ATP‐dependent nucleosome remodelers act as the molecular acrobats of the chromatin accessibility landscape. We highlight that the three CHD subfamilies represent a developmental division of labor: subfamily I maintains pluripotent chromatin accessibility, subfamily II drives lineage commitment through chromatin closure, and subfamily III establishes tissue‐specific enhancer and promoter accessibility during organogenesis. By dynamically transforming chromatin architecture through nucleosome sliding, spacing, and ejection, CHD remodelers license the transitions between cell states, ensuring that the epigenetic landscape is reshaped for the specific needs of a stem cell today and a differentiated cell type tomorrow. We discuss how disruption of this licensing, through mutation or loss of family members, leads to severe neurodevelopmental disorders, including CHARGE syndrome, autism spectrum disorder, and intellectual disability. We close by raising outstanding questions including how CHD activity is regulated, how co‐expressed paralogs coordinate their activities, and whether CHD dysfunction can be therapeutically targeted.

## Graphical abstract

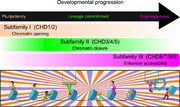

## 1 Introduction

The eukaryotic nucleus is a densely packed environment where DNA, histones, transcription factors, and RNA molecules compete for space. ATP‐dependent nucleosome remodelers must perform intricate mechanical maneuvers in this tight micro‐environment, without compromising DNA integrity while dynamically modulating gene expression to meet the cell's physiological needs. This mechanical agility is essential for addressing a fundamental question in biology: how do cells with identical genomes give rise to vastly different cell types?

**FIGURE 1:**
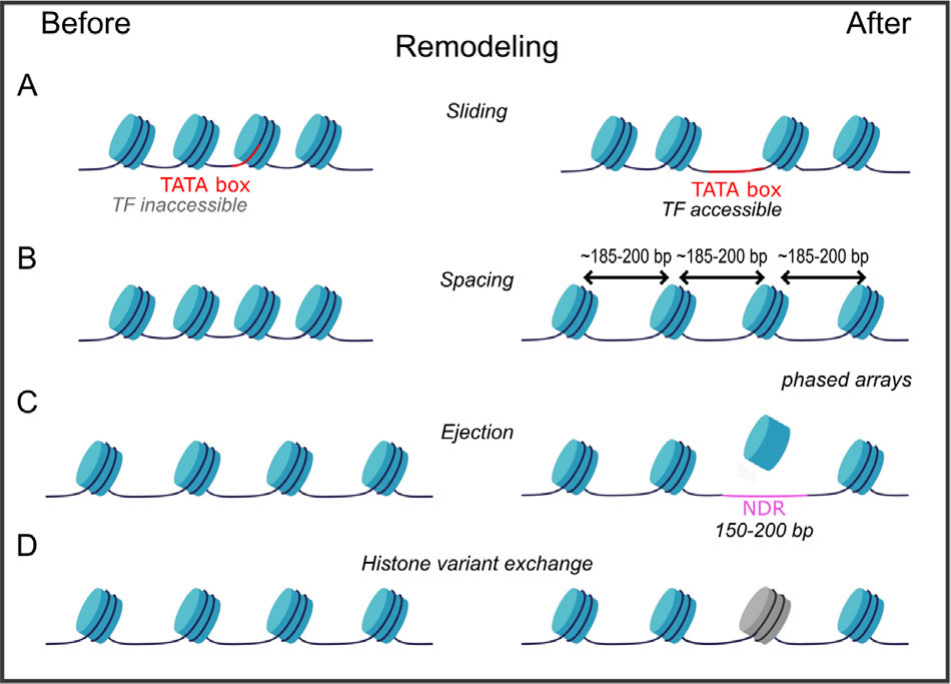
ATP‐dependent nucleosome remodeling mechanisms. Schematic overview of the four primary activities of ATP‐dependent nucleosome remodelers. (A) Sliding: the remodeler translocates DNA relative to the histone octamer, repositioning the nucleosome along the DNA to expose or occlude DNA regulatory elements. (B) Spacing: remodelers translocate DNA to establish regular, evenly spaced nucleosome arrays across gene bodies by repositioning nucleosomes at defined intervals following the passage of RNA Polymerase II. This periodic organization facilitates elongation, maintains chromatin integrity, and prevents cryptic transcription initiation from internal genic promoters. (C) Ejection: complete removal of the histone octamer creates nucleosome‐depleted regions (NDRs) at active DNA regulatory elements, providing access for transcription factors and the transcriptional machinery. (D) Histone variant exchange: INO80 family remodelers catalyze exchange between canonical histone H2A and its variant H2A.Z, altering nucleosome stability, DNA accessibility, and chromatin function at regulatory elements (See also Box 1). Chromatin structures created with BioRender.com.

The answer lies not in the genetic code itself, but in the regulatory machinery controlling genomic accessibility at the nucleosome level. Nucleosomes, 147 bp of DNA wrapped ∼1.7 times around eight histone proteins (octamers) often occlude DNA regulatory elements (promoters and enhancers) from transcription factor binding [1, 2, 3, 4, 5, 6]. Nucleosome remodelers, which fall into four major subfamilies (CHD, SWI/SNF, ISWI, INO80), hydrolyze ATP to dynamically reorganize nucleosomes and alter the accessibility landscape to either promote or prevent DNA‐templated activities (Figure 1). Remodelers dynamically reshape this landscape through three primary activities: sliding (repositioning nucleosomes along DNA), spacing (organizing nucleosomes into regular arrays), and ejection (eviction; removing histone octamers to create nucleosome‐free DNA; Box 1) [7, 8, 9, 10]. Different remodeler families specialize in distinct activities: CHD and ISWI remodelers principally drive nucleosome sliding and spacing while SWI/SNF remodelers are primarily associated with nucleosome sliding and ejection. CHD remodelers can also contribute to nucleosome disassembly and reassembly, a process known to facilitate ongoing transcription, in cooperation with histone chaperones [11, 12, 13]. A fourth activity, histone variant exchange, is performed primarily by the INO80 family of remodelers [14, 15, 16]. Without continuous remodeling, genetic information remains physically inaccessible even when transcription factors are present.

While the other major nucleosome remodeling families, SWI/SNF, ISWI, and INO80, have been comprehensively reviewed [7, 17, 18, 19, 20, 21, 22], the CHD family has received comparatively less systematic attention. Building on previous overviews of CHD proteins in development and disease [23, 24, 25, 26], here we synthesize the CHD remodelers as a coherent functional system, orchestrating an epigenetic choreography from pluripotency to organogenesis.

**Three major remodeling activities of CHD family proteins**

*Sliding*: Repositioning nucleosomes exposes DNA regulatory elements that control transcription initiation and other DNA‐templated processes. A classic example is the TATA box, a highly conserved 5'‐TATAAA‐3' promoter element often located 25–30 bp upstream of transcription start sites (TSSs) [27, 28]. When a nucleosome occludes the TATA box, TATA‐binding protein (TBP) and general transcription factors (GTFs) cannot assemble, and RNA Polymerase II cannot be recruited. By sliding the nucleosome away, remodelers expose the promoter, allowing the pre‐initiation complex (PIC) to form and transcription to begin [29].

Structural studies of yeast Chd1 and human CHD4 bound to a nucleosome revealed that their ATPase motor engages DNA at superhelical location 2 (SHL2), generating a translocation‐competent state that drives nucleosome sliding along DNA [30, 31]. At the single‐molecule level, Chd1 dynamically shifts nucleosomes back and forth [32]. CHD1 can also slide noncanonical nucleosome substrates including hexasomes (nucleosomes lacking one H2A/H2B dimer) which arise during active transcription, suggesting that CHD sliding activity is adapted to the dynamic chromatin landscape encountered during elongation [33].

*Spacing*: CHD remodelers establish regular, evenly spaced nucleosome arrays that define chromatin architecture [23, 34, 35, 36]. In yeast, the nucleosome repeat length is ∼165 bp (147 bp of nucleosomal DNA plus ∼20 bp linker DNA), whereas in mammals it is longer, typically 185–200 bp [37, 38, 39, 40]. Unlike random nucleosome positioning, this phased structure enables the smooth processivity of RNA Polymerase II through gene bodies, prevents cryptic transcription initiation from internal genic promoters and ensures efficient RNA Polymerase II elongation [41]. Spacing is particularly critical during differentiation, when CHD remodelers must coordinately reorganize large genomic regions to establish new transcriptional programs.

*Ejection (eviction)*: Completely removing histone octamers creates accessible DNA. Active genes require stable, nucleosome‐depleted regions (NDRs) that are 150–200 bp stretches of nucleosome‐free DNA at their promoters to accommodate the transcriptional machinery [40]. NDRs are a conserved feature of active promoters from yeast to humans, underscoring the evolutionary importance of maintaining accessible regulatory DNA [42]. Without continuous remodeler activity, including contributions from CHD family members, histones reassociate with DNA, re‐occupying NDRs and silencing genes [43]. Thus, maintaining open chromatin at active promoters is an ATP‐dependent, energy‐consuming process. CHD remodelers can function in concert with histone chaperones such as Nap1 or FACT to facilitate these disassembly and reassembly processes, ensuring that nucleosomes are removed during transcription but restored to maintain genome stability [11, 13, 44, 45, 46].

CHD‐mediated nucleosome ejection is crucial for both oncogenic and housekeeping transcriptional program. CHD6 provides a compelling example where it disrupts nucleosomes in promoters and gene bodies, resulting in the transcriptional activation of oncogenic pathways [47, 48].

Nucleosome ejection by CHD remodelers goes beyond transcriptional regulation in somatic cells. In the post‐meiotic phase of sperm development, nucleosomes must be globally replaced with protamines (sperm‐specific proteins that can tightly compact the parental genome). CHD5 acts as a master regulator of this histone‐to‐protamine process [49]. These studies reveal that nucleosome ejection by CHD remodelers operates across vast biological contexts.

## 2 CHD Protein Core Architecture

### 2.1 SNF2 ATPase: The Evolutionary Engine

**FIGURE 2:**
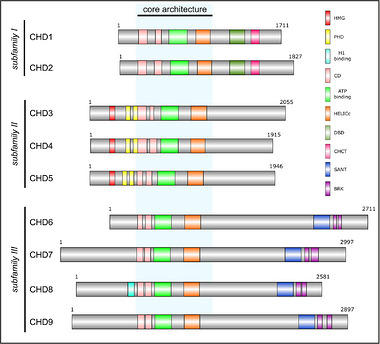
Domain architecture of the mammalian CHD protein family. Schematic representation of the nine mammalian CHD proteins (CHD1‐CHD9), organized by subfamily and aligned at their tandem chromodomains to highlight the conserved core and the divergence of subfamily‐specific domains on either side. All members share a core domain architecture composed of tandem chromodomains (CD) and SNF2‐type ATPase domain comprising the ATP‐binding and HELICc helicase C‐terminal sub‐domains. Subfamily I remodelers (CHD1 and CHD2) additionally contain a C‐terminal DNA‐binding domain (DBD) and a CHD C‐terminal domain (CHCT). Subfamily II remodelers (CHD3‐CHD5) contain two N‐terminal plant homeodomain zinc fingers (PHD) and an N‐terminal HMG box‐like domain present across all three members. Subfamily III remodelers (CHD6‐CHD9) contain a SANT domain and tandem BRK domains C‐terminal to the ATPase core, as well as variable N‐terminal regions that mediate distinct protein interactions and confer functional specificity among individual members. An H1‐binding domain is indicated for CHD8. Domain sizes and relative positions are drawn to approximate scale. Figure drawn using IBS (Illustrator for Biological Sequences) [161].

Every remodeler protein, including the nine CHDs in mammals, possesses a SNF2‐type ATPase domain, which acts as the core catalytic engine of nucleosome remodeling (Figure 2) [50]. The name SNF2 derives from the Sucrose Non‐Fermenting 2 (SNF2) gene, first identified in *Saccharomyces cerevisiae* [51]. Belonging to the helicase Superfamily 2, SNF2‐type proteins evolved as DNA translocases rather than strand‐separating helicases [52]. Like an acrobat performing a hand‐over‐hand traverse along a rope, the SNF2 motor grips and pulls DNA through its active site, generating the mechanical force needed to shift DNA relative to the histone octamer [30, 53, 54].

Structurally, this engine comprises two RecA‐like lobes (∼150–200 amino acids each): the DExx (helicase ATP‐binding) and HELICc (helicase C‐terminal) domains, with ATP binding and hydrolysis occurring at their interface. This ATP‐driven conformational change produces a ratcheting motion that translocates DNA in discrete steps at a specific internal site on the nucleosome SHL2, generating a wave of movement that shifts the histone octamer to a new position [55, 56, 57, 58].

### 2.2 Chromodomains Meet SNF2

The chromodomain, named for its role in chromatin organization modification, was identified through studies of transcriptional repression in *Drosophila melanogaster* [59, 60]. Heterochromatin Protein 1 (HP1) and Polycomb (Pc) were the first chromodomain‐containing proteins described, which bind methylated lysine residues on histone H3 tails to establish and maintain silent chromatin states [61, 62, 63, 64]. These findings established the paradigm that specialized protein domains can read histone modification patterns and translate them into transcriptional consequences. CHD proteins integrate this reading capability directly with ATP‐dependent nucleosome remodeling, coupling epigenetic recognition to chromatin restructuring within a single protein.

All CHD proteins contain tandem chromodomains N‐terminal to the SNF2 ATPase (Figure 2). Except for CHD2, each chromodomain (∼50 amino acids) of mammalian CHD family members forms a functional aromatic cage that binds methylated lysines on histone tails with context‐dependent specificity. For example, MLL/COMPASS deposits H3K4me3 at active genes, which CHD1 can bind [65]. The tandem architecture amplifies specificity through avidity effects, as the two chromodomains together achieve higher binding affinity and selectivity than either would alone [66]. Together, histone marks and chromodomain recognition create layers of chromatin regulation: histone marks recruit CHD remodelers to specific regions where they actively remodel the epigenetic landscape to modulate cell‐type‐specific transcription.

Having established how CHD proteins read and respond to histone modifications through their chromodomain‐SNF2 architecture, we examine how this mechanism operates across development in the following sections. We discuss CHD subfamily diversification: subfamily I (CHD1/2) maintaining pluripotent chromatin, subfamily II (CHD3/4/5) establishing lineage commitment through chromatin closure, and subfamily III (CHD6/7/8/9) organizing neuronal landscapes.

## 3 Subfamily I: Guardians of Pluripotency

CHD1 is highly expressed in embryonic stem cells (ESCs), where it functions as a global chromatin opener [67, 68, 69]. The stem cell epigenome is characterized by greater chromatin accessibility compared to differentiated cells, and CHD1 is critical for maintaining this open state. Loss of CHD1 triggers spontaneous differentiation and widespread chromatin compaction [69]. CHD1's chromodomains recognize H3K4me3, promoting remodeler occupancy over active loci including pluripotency regulators *Oct4*, *Sox2*, and *Nanog* [66]. By maintaining accessibility at these master regulator genes, CHD1 demonstrates that the pluripotent state is not passive but requires continuous ATP‐dependent nucleosome remodeling.

Beyond promoters, CHD1 plays a critical role in gene body chromatin organization. CHD1 associates with the elongation complex Paf1C through its C‐terminal CHCT domain and organizes nucleosome spacing across transcribed regions [70, 71, 72, 73]. This spacing ensures chromatin integrity during elongation and prevents cryptic transcription initiation within gene bodies, demonstrating that CHD1 acts throughout the entire transcription cycle [41].

**FIGURE 3:**
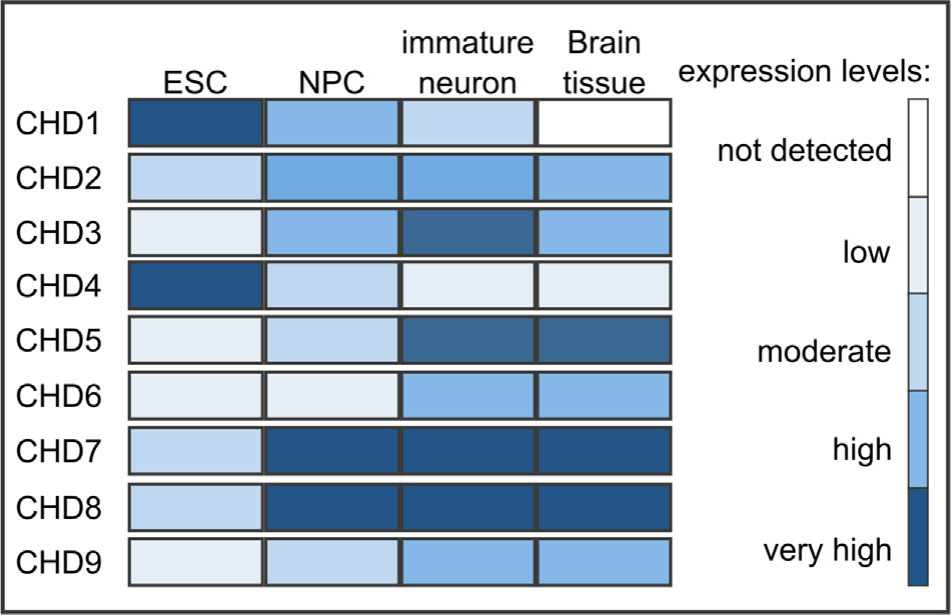
Expression dynamics of CHD family members across neural lineage progression. Heatmap depicting the relative expression levels of all nine CHD proteins (CHD1–CHD9) across four stages of neural development: embryonic stem cells (ESCs), neural progenitor cells (NPCs), immature neurons, and brain tissue. Expression levels are represented on a semi‐qualitative scale from not detected (white) through low, moderate, and high to very high (dark blue), compiled from multiple RNA‐seq studies [162, 163, 164]. The heatmap illustrates the dynamic and stage‐specific expression of CHD subfamily members during the transition from pluripotency to differentiated neural cell types, reflecting the sequential handoff of chromatin regulatory functions.

Compared to CHD1, CHD2 is less characterized; however, its expression overlaps with CHD1 in ESCs but extends into neural progenitor populations (Figure 3), demonstrating a role in bridging the pluripotent state to neural commitment, which is regulated by CHD subfamilies II and III [74, 75, 76, 77, 78]. Unlike CHD1, which directly recognizes H3K4me3 through its chromodomains, CHD2 displayed weaker affinity to methylated histones due to an extension in both chromodomains [79]. Therefore, CHD2 is thought to be recruited to chromatin primarily through interactions with transcription factors and the elongation machinery. While CHD1 and CHD2 maintain open chromatin for pluripotency transcription factors, neural commitment requires the silencing of OCT4/NANOG/SOX2 and the activation of neural determinants such as PAX6 [80]. This transition demands active chromatin closure to reorganize the genetic playground. Subfamily II CHDs provide this opposing function.

## 4 Subfamily II: Chromatin Closure and Lineage Commitment

Subfamily II members (CHD3, CHD4, and CHD5) differ from the subfamily I architecture by adding a high mobility group (HMG) and two lant homeodomain zinc fingers (PHDs) N‐terminal to the chromodomains and SNF2 ATPase core and losing the C‐terminal CHCT domain (Figure 2) [26].

HMG domains (∼80 amino acids) were originally identified as sequence independent DNA binding factors [81, 82]. HMGs have a capacity to bend and distort the double stranded DNA to facilitate transcription factor binding and nucleosome remodeling at DNA regulatory elements [83, 84]. However, recent structural insights suggest that the CHD4 HMG‐like domain has evolved specialized regulatory functions beyond simple DNA distortion [23]. CHD4 HMG has a higher affinity to poly(ADP‐ribose) (PAR) than to DNA [34]. PAR is synthesized by PARP1 at sites of DNA strand breaks, serving as a DNA damage signal [85]. This positions the HMG‐like domain as a dual‐function module contributing to remodeling activity under normal conditions while also enabling CHD4 to be recruited to damaged chromatin through PAR recognition [23, 34].

PHD fingers (∼60 amino acids) were initially discovered in *Arabidopsis thaliana* [86, 87] and later found to be conserved throughout eukaryotes. Structural NMR and in vitro histone peptide binding assays established that PHD fingers of subfamily II can read histone modifications such as H3K9ac or repressive marks such as H3K9me2/3 [88, 89, 90, 91]. Interestingly, PHD fingers can bind unmodified H3K4 in some contexts [92, 93, 94, 95]. This functional versatility established PHD fingers as tunable epigenetic sensors capable of distinguishing between transcriptionally active and silenced chromatin states. However, how PHD fingers contribute to CHD chromatin targeting and activity in vivo is an open and important question. Integrating these domains into a dual reading system (chromodomains plus PHD fingers) likely enables subfamily II CHDs to interpret more complex chromatin states than subfamily I. Additionally, while subfamily II CHDs are able to act alone, they also function as catalytic subunits within multi‐protein complexes, integrating nucleosome remodeling with histone deacetylation and other regulatory activities.

Most prominently, subfamily II CHDs serve as the ATPase subunits of the nucleosome remodeling and deacetylase complex (NuRD), which couples nucleosome repositioning with histone deacetylation [96, 97, 98]. This coordination creates a chromatin‐closing mechanism: where histone deacetylase (HDAC)‐mediated deacetylation and nucleosome sliding work in tandem to enforce transcriptional silencing. However, NuRD is increasingly viewed as a versatile complex, characterized by a modular architecture that allows for context‐specific subunit exchange and functional plasticity [99]. Furthermore, CHD4, as part of the ChAHP complex (CHD4‐ADNP‐HP1), represses transposable elements and lineage‐specifying genes through a locally restricted, HDAC‐independent chromatin‐closing mechanism, and in some contexts acts independently of either complex entirely [100, 101, 102].

### 4.1 CHD4: Reorganizing the Genetic Playground for Neural Identity

In ESCs, CHD4 acts in opposition to CHD1 [103, 104]. While CHD1 maintains the open chromatin landscape characteristic of pluripotency [105], CHD4‐containing NuRD complexes are recruited to lineage‐specific genes that must remain silent. During neural differentiation, this opposing relationship becomes critical: the CHD4/NuRD complex is recruited to the very pluripotency loci that CHD1 keeps accessible and actively closes them through nucleosome repositioning and histone deacetylation. Simultaneously, CHD4/NuRD reinforces the silencing of nonneural lineage genes, such as mesodermal and endodermal fate determinants. Loss of NuRD results in retention of pluripotency gene expression and failure to undergo lineage commitment, demonstrating that neural specification requires NuRD‐mediated chromatin closure to override the open state maintained by CHD1 [104, 106].

### 4.2 CHD3 And CHD5: Consolidating the Neural Program

CHD3 and CHD5 extend subfamily II's chromatin‐closing function into later stages of neural development (Figure 3). CHD3 is highly expressed in neural progenitors, where it silences alternative fates [107], while CHD5 is enriched in post‐mitotic neurons, maintaining the differentiated state by preventing reactivation of progenitor genes [108, 109]. While subfamily II CHDs establish and maintain silenced chromatin states, the proper activation of tissue‐specific gene expression programs during organogenesis requires an additional level of regulation: the remodeling of DNA regulatory elements and the integration of transcription factor signaling with chromatin accessibility. Subfamily III CHDs provide this capability.

## 5 Subfamily III: Neurodevelopmental Regulators

Subfamily III represents the most structurally complex group of CHD proteins (Figure 2). Beyond the chromodomain‐SNF2 core, subfamily III members contain SANT and BRK domains C‐terminal to the SNF2 ATPase that enable specialized functions during development [110, 111, 112, 113]. The SANT domain (∼50 amino acids; named after SWI3, ADA2, N‐CoR, and TFIIIB) facilitates non‐sequence specific DNA binding and histone tail interactions, enabling recognition of chromatin contexts beyond methylation marks alone [110, 111, 114]. The BRK domain (∼100 amino acids; named after *D. melanogaster* Brahma and Kismet) mediates protein–protein interactions with transcription factors and coactivators, enabling direct recruitment to target loci [112, 113, 115].

Within subfamily III itself, individual members further diverge through N‐terminal sequence variations that mediate unique proteinprotein interactions and confer functional specificity [116]. Together, these domains enable subfamily III CHDs to integrate distinct transcription factor signaling with nucleosome remodeling which is important for the development of unique neuronal subtypes (Figure 2).

### 5.1 CHD7: Enhancer Regulation and CHARGE Syndrome

CHD7 is arguably the most clinically studied among subfamily III due to its central role in human development. Heterozygous loss‐of‐function mutations in *CHD7* cause CHARGE syndrome (an acronym for Coloboma, Heart defects, Atresia of the choanae, Restriction of growth and development, Genital abnormalities, and Ear abnormalities), a severe developmental disorder that affects multiple organ systems [117, 118, 119]. The pleiotropic nature of CHARGE syndrome reflects CHD7's broad expression during embryogenesis and its critical function in establishing tissue‐specific gene expression programs (Figure 3).

CHD7's dual function at promoters and enhancers depends on its integration with distinct histone modifications; it colocalizes with H3K4me3 at promoters in stem cells and H3K4me1 at active tissue‐specific enhancers in neural progenitor cells [120, 121, 122]. This flexibility in mark recognition illustrates how subtle differences in chromatin reading underlie the multifunctional roles of subfamily III members [123].

During neural crest development, CHD7 cooperates with the PBAF complex to establish accessibility at enhancers controlling neural crest specifier genes [120]. In CHD7‐deficient cells, these enhancers fail to become accessible, and neural crest cells cannot properly differentiate into their diverse derivatives, including neurons, glia, and craniofacial mesenchyme [120].

Beyond the neural crest, CHD7 plays critical roles in sensory organ development (inner ear, olfactory epithelium, and retinal neurogenesis), heart morphogenesis, and hypothalamic‐pituitary axis formation [124, 125, 126, 127]. ChIP‐seq studies of CHD7 across these tissues reveal thousands of tissue‐specific binding sites, predominantly at enhancers rather than promoters [121, 128]. This enhancer‐centric localization distinguishes CHD7 from CHD1 (promoter‐focused) and CHD4 (both promoters and enhancers), highlighting functional specialization within the CHD family.

### 5.2 CHD8: Promoter Regulation and Autism Spectrum Disorder (ASD)

CHD8 is one of the highest‐confidence ASD risk genes, with *de novo* loss‐of‐function mutations identified in ∼0.5% of ASD cases [129, 130, 131]. CHD8 is highly expressed in neural progenitors and developing neurons, where it regulates genes involved in nucleosome remodeling, cell cycle regulation, and neuronal differentiation.

While CHD8 occupies both enhancers and promoters, it shows strong enrichment at actively transcribed gene promoters marked by H3K4me3, particularly those involved in neurodevelopmental processes [132, 133]. At the same time, it can exert context dependent repression by interacting with p53 and β‐catenin [134, 135] through its N‐terminus, which is distinct from the chromodomain module, to recruit histone H1 and repress transcription at selected target genes (Figure 2). Haploinsufficiency of CHD8 in mice and human neural organoids leads to aberrant proliferation of neural progenitors, altered neuronal migration, and macrocephaly (enlarged brain) [136, 137] which are features observed in many CHD8‐mutant ASD patients [138, 139, 140].

Mechanistically, CHD8 occupies promoters and gene bodies of neuronal genes [132, 133], where it likely maintains proper chromatin organization to ensure efficient transcriptional elongation. Loss of CHD8 leads to reduced expression of genes involved in synapse formation, axon guidance, and neural connectivity [132, 140]. Interestingly, CHD8 also regulates the expression [137, 141] and activity [142] of many other nucleosome remodelers, transcription factors, and histone modifiers, positioning it as a central coordinator that organizes chromatin state across the neuronal chromatin landscape. This network‐level function explains the profound neurodevelopmental consequences of CHD8 mutations where loss of this single remodeler cascades into dysregulation of many others.

### 5.3 CHD6 and CHD9: Emerging Roles in Neural and Stem Cell Biology

While less characterized than CHD7 and CHD8, recent studies have begun to describe the functions of CHD6 [143] and CHD9 [144], revealing that every subfamily III member contributes to development.

CHD6 is highly expressed in neural tissues and hematopoietic cells [145]. Recent work has implicated CHD6 in the DNA damage response and in maintaining genome stability during neural development. CHD6 appears to be recruited to oxidative‐stress‐damaged DNA sites through a process initiated by its N‐terminus (distinct from other subfamily III members) and strengthened by its chromodomains, where it promotes cell survival through antioxidant transcriptional responses and proper chromatin organization [146]. CHD6 interacts with initiating and elongating RNA polymerase II [143], suggesting that CHD6 facilitates transcriptional output likely to regulate DNA damage response upon oxidative stress [147].

While CHD9 is expressed in the developing brain and identified in neural stem cells [148], its specific developmental contributions appear more subtle than those of its subfamily peers. Unlike the embryonic lethality seen in *Chd7* or *Chd8* mutants, *Chd9* knockout mice are viable and fertile, suggesting that CHD9 may serve as a redundant or context‐specific modulator rather than a primary driver of development [149]. Interestingly, CHD9 has also been implicated in mesenchymal stem cell differentiation, specifically osteogenic lineage where it regulates the expression of important factors including Runx2 and osteocalcin [144], hinting at broader functions beyond the nervous system that remain to be fully explored.

## 6 Stable Cell Identity Through Dynamic Chromatin Maintenance

The developmental journey from pluripotent stem cell to differentiated neuron illustrates a fundamental principle: cell identity requires stable chromatin architecture maintained through continuous dynamic remodeling. Like molecular acrobats who must constantly adjust to maintain balance, CHD remodelers continuously reshape chromatin to counteract thermodynamically favored nucleosome reassembly that would compact the genetic playground into an inaccessible state. This paradox, stability through dynamism, explains several key biological phenomena.

(1) Development is largely irreversible [150]. Once CHD4‐containing NuRD complexes silence pluripotency genes during differentiation [103], those loci become progressively inaccessible through reinforcing mechanisms: chromatin compaction, DNA methylation, and loss of active histone marks [151]. The genetic playground, once reorganized for neural identity, cannot spontaneously revert to the pluripotent configuration.
(2) Cellular reprogramming to pluripotency is inherently difficult [152]. Reverting a somatic cell into a pluripotent state (induced stem cells) requires dismantling a chromatin landscape actively maintained by subfamily II complexes.
(3) CHD mutations cause pleiotropic developmental disorders. Disrupting a CHD protein doesn't eliminate a single gene's activity but destabilizes entire chromatin landscapes. Because each CHD protein organizes chromatin accessibility across hundreds or thousands of genes, a single mutation can have cascading effects. The severity of CHARGE syndrome (CHD7) and CHD8‐associated autism reflects genome‐wide failures of chromatin organization where the molecular acrobats can no longer maintain the genetic playground, and development proceeds chaotically.

The principle that chromatin architecture must be actively maintained underscores why CHD proteins are essential developmental regulators and why their disruption has such profound consequences for human disease.

## 7 CHD Mutations in Human Neurodevelopmental Disorders

The critical roles of CHD proteins in neural development are starkly illustrated by human genetics. Mutations in CHD family members cause severe neurodevelopmental disorders, demonstrating that proper nucleosome remodeling is not merely permissive but essential for human brain development (Table 1).

**TABLE 1:** The summary of mammalian CHD family: domain architecture, chromatin activities, and developmental roles with associated human disorders.

CHD Protein	Histone mark target(s)	Primary chromatin activity	Expression/Developmental context	Key developmental role	Associated human disorder(s)
Subfamily I: Chromodomains + SNF2 ATPase + DBD + CHCT					
CHD1	H3K4me3	Nucleosome sliding; nucleosome spacing in gene bodies; NDR maintenance at promoters	Highly expressed in ESCs; broadly expressed	Maintains open chromatin at pluripotency genes (*Oct4*, *Sox2*, *Nanog*); organizes nucleosome spacing during transcription elongation	Pilarowski–Bjornsson syndrome [153, 155] (developmental delay, ID); somatic mutations in prostate cancer [154]
CHD2	Not fully characterized	Nucleosome remodeling at neuronal gene loci (less characterized than CHD1)	ESCs and neural progenitors	May bridge pluripotency (CHD1) and neural commitment (subfamilies II/III); role in neurogenic gene regulation	Epileptic encephalopathy [155]; ID; developmental delay [156]
Subfamily II: HMG + PHD zinc fingers + Chromodomains + SNF2 ATPase; function within NuRD complex					
CHD3	H3K4me0 (unmodified H3); H3K9me3 and H3K9ac	Nucleosome repositioning coupled with histone deacetylation (NuRD complex)	Neural progenitors	Silences alternative neural fates during neural progenitor differentiation	Snijders Blok–Campeau syndrome (ID, macrocephaly, impaired speech) [157]
CHD4	H3K4me0; H3K9me3; H3K9ac	Nucleosome repositioning + histone deacetylation (NuRD); chromatin closure at pluripotency and nonneural lineage genes	ESCs (antagonistic to CHD1); neural progenitors; broadly expressed	Closes chromatin at pluripotency loci during differentiation; silences nonneural lineage genes; essential for lineage commitment	Sifrim–Hitz–Weiss syndrome (ID, congenital heart defects, hypogonadism) [158, 159]
CHD5	H3K4me0	Chromatin closure and maintenance of silenced states in post‐mitotic neurons	Post‐mitotic neurons; enriched in mature neural tissue	Maintains differentiated neuronal state by preventing reactivation of progenitor genes; tumor suppressor function	ID and developmental delay (rare variants); CHD5 deletion linked to neuroblastoma (tumor suppressor) [108, 109]
Subfamily III: Variable N terminus + Chromodomains + SNF2 ATPase + SANT domain + BRK domain					
CHD6	Not fully characterized	Nucleosome remodeling at DNA damage sites	Neural tissues; hematopoietic cells	DNA damage response and genome stability during neural development; neural stem cell self‐renewal (CHD1‐like function in neural lineages)	Hallermann–Streiff syndrome (craniofacial and growth abnormalities); emerging links to ID [147]
CHD7	H3K4me3 (promoters in stem cells); H3K4me1 (enhancers in neural progenitors)	Establishes accessibility at tissue‐specific enhancers; also active at promoters in stem cells; enhancer‐centric localization	Broadly expressed during embryogenesis; neural crest: inner ear, heart, hypothalamic‐pituitary axis	Establishes tissue‐specific enhancer accessibility during organogenesis; critical for neural crest specification, sensory organ development, heart morphogenesis	CHARGE syndrome [118, 160]
CHD8	H3K4me3 (promoters)	Maintains nucleosome positioning at promoters and gene bodies of neuronal genes; promoter‐centric localization	Neural progenitors; developing neurons; highly expressed during cortical neurogenesis	Regulates transcription of neuronal differentiation, cell‐cycle, and nucleosome remodeler genes; central coordinator of the neuronal epigenome	ASD: de novo LoF mutations in ∼0.5% of ASD cases; macrocephaly, ID, GI issues; one of highest‐confidence ASD risk genes [130, 131]
CHD9	Not fully characterized	Nucleosome remodeling at neuronal gene promoters; colocalizes with CHD8 at H3K4me3‐marked promoters	Developing brain; oligodendrocyte precursors; mesenchymal cell differentiation	Roles in oligodendrocyte differentiation and myelination; potential cooperative function with CHD8 at neuronal promoters; metabolic regulation	ID (emerging evidence); full clinical spectrum not yet defined

*Note 1*: Histone mark targets listed reflect current evidence from ChIP‐seq and in vitro binding studies. “Not fully characterized” indicates that systematic binding specificity studies have not yet been published.

*Note 2*: All CHD proteins share a conserved SNF2‐type ATPase domain (DExx + HELICc lobes) and tandem chromodomains. Only subfamily‐distinguishing and member‐specific accessory domains are highlighted.

*Abbreviations*: ID, intellectual disability; LoF, loss of function; GI, gastrointestinal; NDR, nucleosome‐depleted region; ESC, embryonic stem cell; NuRD, nucleosome remodeling and deacetylase complex, ASD, Autism spectrum disease.

Across all CHD‐associated disorders, the unifying pathology is failed chromatin landscape organization during development. These are not simply “gene expression” defects but failures to properly reorganize chromatin: to maintain it open (CHD1/2), close it appropriately (CHD3/4/5), or establish tissue‐specific accessible regions (CHD6/7/8/9). Human genetic data validate what molecular studies predict: that CHD proteins are essential architects of chromatin accessibility whose coordinated activity is required for normal development. The genetic playground, without its molecular acrobats, cannot support the complex chromatin choreography of human brain development.

## 8 Future Perspectives

The CHD family exemplifies a fundamental principle of developmental biology: complex organisms require sophisticated chromatin regulation. The evolution of CHD proteins from *Chd1*, the sole CHD protein found in *S. cerevisiae*, to nine elaborate multi‐domain CHD remodelers in mammals mirrors the increasing complexity of metazoan development. Yet despite decades of research, fundamental questions about how these molecular acrobats execute their chromatin choreography remain unanswered.

*How is CHD activity regulated?* While we understand that chromodomains target CHDs to methylated histones, the regulatory logic governing what signals activate or suppress remodeling activity at specific loci, beyond their targeting to histones or DNA, remain poorly characterized. Post‐translational modifications of CHD proteins (phosphorylation, acetylation, ubiquitination) likely modulate activity, but the precise mechanisms remain unknown.

*How do co‐expressed CHD family members coordinate?* Many cell types co‐express multiple CHDs (Figure 3; e.g., neural progenitors express CHD1, CHD2, CHD4, CHD7, and CHD8). Do these proteins compete for nucleosomal substrates, or do they cooperate through division of labor? Emerging evidence suggests partial functional redundancy in some contexts but non‐redundancy in others (as human disease demonstrates). Understanding the rules governing CHD coordination and potential compensation remains a critical gap.

*What determines remodeling outcome?* The same CHD protein can slide, space, or eject nucleosomes depending on context. What molecular features (nucleosome stability, flanking DNA sequence, transcription factor occupancy, or chromatin compaction state) determine which outcome occurs? Answering this will require single‐molecule approaches that can visualize CHD proteins remodeling individual nucleosomes in real time.

*Can CHD dysfunction be targeted therapeutically?* The pleiotropic effects of CHD mutations and their ubiquitous expression make direct therapeutic targeting challenging. However, several strategies merit exploration: small molecules that modulate CHD‐transcription factor interactions in disease contexts, leveraging functional redundancy to upregulate compensatory CHD family members, or targeting downstream effectors (HDACs, methyltransferases) whose modulation might partially restore chromatin organization in CHD‐deficient cells.

*Can CHDs be utilized as therapeutic agents?* Recent perspectives on “epigenetic rescue” suggest that CHDs may be co‐opted as an alternative gene therapy. Engineered CHDs can be deployed to boost the expression of the functional copy or silence the expression of dominant‐negative mutations in many genetic diseases, offering a way to reprogram the epigenetic network without correcting the genetic mutations.

*How do CHDs cooperate with other remodeler families?* Developmental transitions often require sequential or simultaneous action by multiple remodeler families (e.g., CHD7 and SWI/SNF complexes cooperate at enhancers) [120, 121]. Deciphering the logic of multi‐remodeler collaboration (which family acts first, how they're coordinated, and whether their activities are antagonistic or synergistic) remains an open question essential for understanding complex developmental transitions.

## 9 Conclusions

Chromatin accessibility is not a static feature of the genome but an actively maintained state requiring continuous energy expenditure. As technologies advance (single‐cell chromatin accessibility profiling, live‐cell imaging of remodeler dynamics, and CRISPR‐based epigenome editing), we move closer to understanding not only how individual CHD proteins function, but how their coordinated activity constructs the developmental programs that generate complex organisms.

The CHD family represents a conserved solution to the challenge of dynamically controlling chromatin accessibility across cell fate transitions. From CHD1 maintaining pluripotency through chromodomain‐H3K4me3 recognition, to CHD7 orchestrating complex enhancer landscapes during organogenesis, these molecular acrobats continuously reshape the genetic playground to meet the changing demands of development. Understanding their coordinated choreography is essential for both deciphering normal development and treating the disorders that arise when nucleosome remodeling fails.

## Author Contributions

**İsa Özdemir**: writing, reviewing, and editing. **Sarah J. Hainer**: funding acquisition, writing, reviewing, and editing. Both authors approved the final article.

## Conflicts of Interest

The authors declare no conflicts of interest.

## Data Availability

Data sharing is not applicable to this article as no new data were generated or analyzed for this study.
